# Modeling of the Van Der Waals Forces during the Adhesion of Capsule-Shaped Bacteria to Flat Surfaces

**DOI:** 10.3390/biomimetics6010005

**Published:** 2021-01-08

**Authors:** Fathiah Mohamed Zuki, Robert G. J. Edyvean, Hamed Pourzolfaghar, Norherdawati Kasim

**Affiliations:** 1 Department of Chemical Engineering, University of Malaya, Kuala Lumpur 50603, Malaysia; h_pourzolfaghar@yahoo.com; 2 Department of Chemical and Biological Engineering, University of Sheffield, Newcastle Street, Sheffield S1 3JD, UK; r.edyvean@sheffield.ac.uk; 3 Department of Chemistry and Biology, Center for Foundation Studies, National Defence University of Malaysia, Kem Sungai Besi, Kuala Lumpur 57000, Malaysia; herdawati@upnm.edu.my

**Keywords:** bacterial adhesion, capsule-shaped bacteria, pseudomonas putida, surface energy, van der Waals forces

## Abstract

A novel model is developed to evaluate the van der Waals (vdW) interactions between a capsule shaped bacterium (*P. putida*) and flat minerals plates in different approach profiles: Vertically and horizontally. A comparison of the approaches to the well-developed spherical particle to mineral surface (semi-infinite wall and spherical) approach has been made in this investigation. The van der Waals (vdW) interaction potentials for a capsule-shaped bacterium are found using Hamaker’s microscopic approach of sphere to plate and cylinder to plate either vertically or horizontally to the flat surface. The numerical results show that a horizontal orientated capsule shaped bacterium to mineral surface interaction was more attractive compared to a capsule shaped bacterium approaching vertically. The orientation of the bacterial approaching a surface as well as the type and topology of the mineral influence the adhesion of a bacteria to that surface. Furthermore, the density difference among each type of bacteria shape (capsule, cylinder, and sphere) require different amounts of energy to adhere to hematite and quartz surfaces.

## 1. Introduction

Microbes or microorganisms have a robust propensity to attach to a surface [1]. As soon as the microbes associate to a surface, a multistep procedure initiates from initial attachment to eventual formation of a complex community [2]. This complex community (a biofilm) consists of the microbes, both those initially attached and their offspring, embedded in a secretion of an extracellular matrix and can eventually include different microbial species and a variety of solid particles/debris. Biofilms can be either advantageous or have harmful consequences and disadvantageous properties depending on the environment [3]. A better understanding of microbial interactions at surfaces offers opportunities for industrial developments, such as bioremediation [4], treatment of hazardous waste sites [5], bio-filtration [6], and forming bio-barriers to protect soil and groundwater from contamination [7,8,9]. Furthermore, novel cleaning strategies, based on an understanding of how bacteria attach, grow, and detach, are urgently needed by many industries [10,11]. Introducing bacteria into groundwater containing minerals may lead to differences in the attachment of the bacteria onto different mineral surfaces depending on their interaction potentials [12], an aspect that can be used for both industrial and environmental advantage.

The initial attachment process of a microbe, such as a bacterium, to a surface is governed by at least two types of interaction across the aqueous phase. These are the van der Waals (vdW) and Electrical Double Layer (EDL) interactions [13]. There are numerous publications focusing on the van der Waals (vdW) interaction [14,15,16,17,18]. The van der Waals (vdW) interactions between a colloidal particle and a solid surface as a function of separation distance is important in studying the flow of colloidal emulsions through porous media or fibre filters [19,20,21]. This interaction also plays an essential role in problems of bacterial attachment onto the solid surfaces in biological systems [22]. Furthermore, it plays a critical role in bacteria attachment and biofilm formation onto mineral surfaces in groundwater [23]. The computation of the vdW interaction between a spherical particle and a cylindrical surface had been reported by Yongan and Dongqing [24]. Following the Derjaguin approximation approach, the interaction of a spherical particle to a cylindrical surface can be calculated by assuming the interaction is between a sphere and a finite wall. Yongan and Dongqing also found that the vdW force calculated by this assumption was overestimated due to the large curvature and finite-size effect of the cylinder [24]. The actual vdW interaction between two macroscopic bodies is reduced at large distances because the time it takes for electric fields to propagate from one body to another and back causing the fluctuating electric moments become slightly out of phase, owing to the electromagnetic nature of interaction. The reduction of the vdW interaction is called the retardation effect due to the finite speed of light [25,26]. Montgomery et. al. [27] developed the expression for the van der Waals adhesion forces for a variety of particle shapes interacting with an infinite cylinder by a first principle-based approximate macroscopic model (e.g., sphere to plate interaction). Non-traditional geometries including interactions of spherical shell to infinite cylinder; disk-like particle to infinite cylinder; disk-like particle oriented edgewise to an infinite cylinder; and a deformed slice to infinite cylinder were modeled previously. These interaction models were found to have very high interaction energy and adhesion forces. Many more researchers have contributed to the development of the van der Waals interaction potential model expressions between two macroscopic bodies including Israelachvili [28], Bhattacharjee and Elimielech [29], Tadmor [30], and Bhattacharjee and Sharma [31].

In order to determine the van der Waals interaction potential between bacteria and a mineral surface, bacteria which have a wide range of shapes [32], ranging from spheres to rods and spirals, typically a few micrometers in length, are now being further studied due to their arbitrary shapes and non-traditional geometry. For spherical bacteria to surface interactions, the common mathematical van der Waals interaction energy of sphere to flat surface can be used to calculate the potential energy. However, there are no specific mathematical models yet developed to calculate the van der Waals interaction energy between a capsule-shaped bacterium to a flat surface.

In this survey, a capsule-shaped particle to a flat plate surface interaction model (presenting a bacillus cell and the surface it settles on) is derived from a combination of spherical particle and cylindrical particle to flat surface interaction potentials. Thus, in order to develop a model of capsule-shaped interaction, the derivation of interaction potential for single molecule, spherical particle, and cylindrical particle to flat plate surface interaction using the ‘Ring approach’ (based on Hamaker’s pairwise interactions) is presented. The orientation by which the bacterial cell approaches the flat mineral surface, (horizontally or vertically) is also considered in the van der Waals interaction potential approximation.

## 2. Materials and Methods

### 2.1. Capsule Model

Recently, several researchers have used a well-developed sphere to flat surface interaction equation as a comparison to their experimental results by assuming the bacterial shape is spherical. However, bacteria are found in many different shapes; coccus, rod, spiral, and bacillus or capsule (rod with hemispherical ends) and every shape has its own curve and density that affects the interaction potentials between each bacterium or between bacteria and surfaces. Therefore, the curvature effect developed by Yongan and Dongqing [24], can be applied for bacteria to mineral adhesion studies. Based on the sphere to plate and cylinder to plate, a capsule-like bacterial cell to flat plate mineral surface model can be developed for the two approaching positions; vertical approach and horizontal approach.

### 2.2. Hamaker’s Microscopic Approach

Hamaker [33] determined the van der Waals interaction of two macroscopic bodies by carrying out an integration of all the intermolecular interactions. First, van der Waals interactions between two particles were considered as pairwise inter-particle interactions and tended to be generalized on interactions between large bodies. These large body “macroscopic body” interactions were developed by Hamaker by summation of pairwise interactions over all constituent atoms. The Hamaker approach is widely used because of its simplicity and applicability. General dispersion pair interaction potentials between two interacting molecules separated by a distance *r* can be assumed to be of the power-law form:

(1) $$v\left(r\right)=\;-\frac{C_{d}}{r^{n}}$$

where *C_d_ = 3α*^2^*I/*4(4*πε*_0_)^2^ is the Lifshitz-van der Waals potential coefficient (*α =* polarizability; *I* = certain characteristic potentials of the atom) and *n* depends on the specific potential model used for inter-molecular potential, (for *r* > molecule diameter, *d*, *n* = 6) [34].

#### 2.2.1. Molecule-Plate Interaction Potential

**Figure 1:**
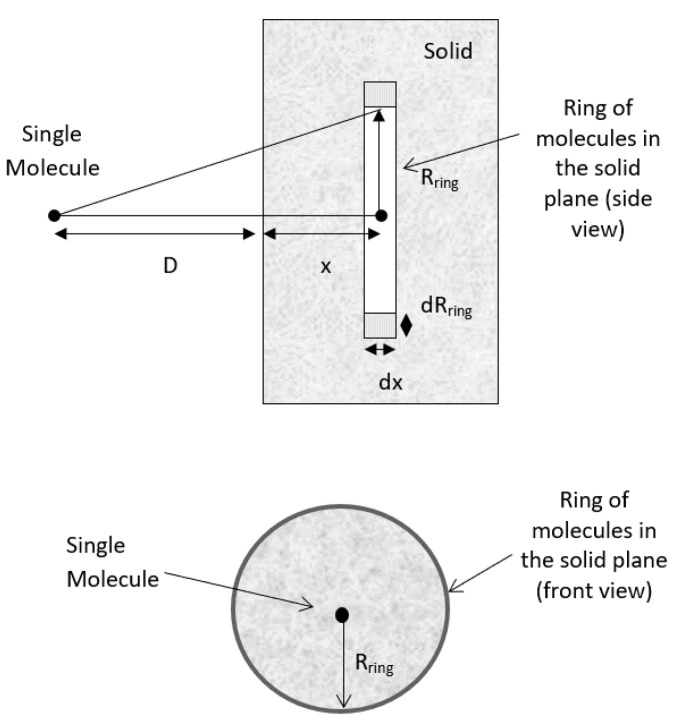
Interaction between a molecule and a solid slab. *D* = distance between the molecule and the surface of the solid plane; *R_ring_* = radius of the ring; *x* is the distance of the ring to the side of the solid; and *r* = distance between the molecule and the ring of molecules in the planar surface.

According to Hamaker’s microscopic approach, the interaction between a molecule and a nearby planar surface of a solid is assumed to be purely attractive. Initially, the volume of the ring containing interacting molecules in the solid is calculated as shown in Figure 1. The radius of the ring, *R_ring_*, and the distance of the ring to the side of the solid, *x*, may be extended to infinity during the integration process to cover all the molecules in the solid slab [35]. Geometrically, the total number of molecules in a circular ring of volume *V = 2πR_ring_dR_ring_dx* is *n = ρ2πR_ring_dR_ring_dx*, where *ρ* is the number density of the molecule (molecule/m^3^). By assuming the sum of the interactions of all molecules present in the solid body with this single molecule, and approximating this summation process with an integral, the net interaction for a molecule of the same material at a distance, *D,* away from the surface of the solid slab becomes:

(2) $$V\left(D\right)=\iint \left[v\left(r\right)\right]\rho 2\pi R_{ring}dR_{ring}dx=-\pi C_{d}\rho \int_{D}^{x=\;\infty }\int_{0}^{R_{ring}}\frac{2R_{ring}dR_{ring}}{r^{6}}\;dx$$

By applying Pythagoras’s theorem, *r*^2^ = (*D* + *x*)^2^ + *R*^2^*_ring_*$r^{2}={\left(D+x\right)}^{2}+R_{\mathrm{ring}}^{2}$$r^{2}={\left(D+x\right)}^{2}+R_{\mathrm{ring}}^{2}$, and since 2*R_ring_dR_ring_* = *dR*^2^*_ring_*, .

and by inserting this into Equation (2), we obtain:

(3) $$V\left(D\right)=\;-\pi C_{d}\rho \int_{D}^{x}\int_{0}^{\infty }\frac{dR_{ring}^{2}}{{\left[{\left(D+x\right)}^{2}+R_{ring}^{2}\right]}^{3}}dx$$

(4) $$V\left(D\right)=-\frac{1}{2}\pi C_{d}\rho \int_{D}^{\infty }\frac{1}{{\left(D+x\right)}^{4}}dx$$

(5) $$V_{vdW}\left(D\right)=-\frac{\pi C\rho }{6D^{3}}$$

From Equation (5), it is shown that the interaction potential energy of a molecule and a macroscopic surface decreases proportionally to *D*^−3^, instead of *D*^−6^ for molecule-molecule interaction. This integration was simplified by several assumptions used by Hamaker: The multibody interactions are discounted and the interactions are only pair-wise; the intervening medium is a vacuum; the molecule and the solid body are not distorted by the attractive forces; the interactions due to the Coulomb forces and permanent dipoles are neglected; all dispersion force attractions are due to a single dominant frequency; the interactions of molecular electron clouds are instantaneous; and the solid body is assumed to have uniform density right to the interface.

#### 2.2.2. Sphere-Flat Plate vdW Interaction Potential

**Figure 2:**
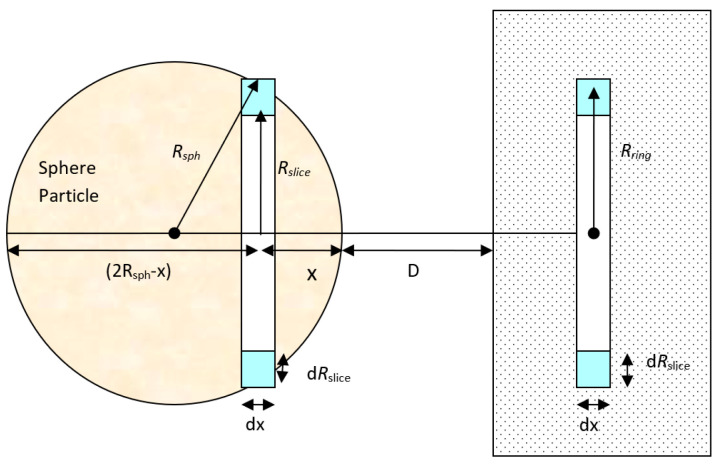
Interaction between a large sphere and a solid flat surface.

By applying a similar procedure for a sphere particle to flat plate, the interaction potential energy between a large spherical particle with radius, *R_sph_*, and the ring in the plate surface (Figure 2) can be calculated. The volume of the circular slice in the sphere is:

(6) $$V=\pi R_{slice}^{2}dx$$

**Figure 3:**
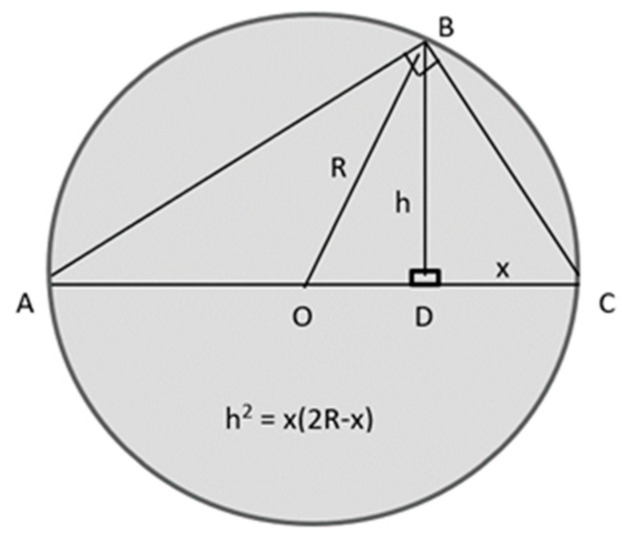
Right-angled triangle used in the proof of the chord theorem of plane geometry.

*R_slice_* is related to *R_sph_* by using chord theorem of plane geometry by applying Pythagoras’s theorem to all triangles in Figure 3. From the figure, [(*AC*)^2^ = (*AB*)^2^ + (*BC*)^2^] giving (*AC*)^2^ = [(*AD*)^2^ + (*BD*)^2^] + [(*BD*)^2^ + (*CD*)^2^]. After simplification and rearrangement, this yields [(2*R*)^2^ = (2*R* − *x*)^2^ + 2*h*^2^ *+ x*^2^] *giving* [*h*^2^ = *x*(2*R* − x)].

Thus, corresponding to Figure 3:

(7) $$R_{slice}^{2}=x\left(2R_{sph}-x\right)$$

Then the total volume of the thin circular slice in the sphere becomes:

(8) $$V=\pi R_{slice}^{2}dx=\pi \left(2R_{sph}-x\right)xdx$$

Therefore, the total number of molecules in this slice is $\pi \rho \left(2R_{sph}-x\right)xdx.$ Since all the molecules in this sphere are at a distance of *(D + x)* from the flat surface, the total sphere to surface dispersion interaction potential can be found by multiplying the number of molecules within the spherical particle and the dispersion interaction potential of a single molecule to a flat surface (Equation (8)) and becomes:

(9) $$V\left(D\right)=-\frac{\pi^{2}C_{d}\rho_{c}\rho_{m}}{6}\int_{x=0}^{x=2R_{sph}}\frac{\left(2R_{sph}-x\right)x}{{\left(D+x\right)}^{3}}dx$$

Therefore, by derivation of this equation (Equation (9)), the van der Waals, vd*W* interaction potential of a large spherical particle can be calculated. This equation can then be applied into the calculation of the vd*W* interaction potential for the capsule-shaped particle to flat plate interaction. The capsule model is defined by the combination of a spherical and a cylindrical-shaped particle.

#### 2.2.3. Cylinder to Flat Plate vdW Interaction Potential

Once the spherical particle to flat surface van der Waals, vd*W* interaction potential has been derived, the vd*W* interaction potential for a cylindrical particle to flat plate interaction can be derived in order to justify and satisfy the suggested capsule model. The vd*W* interaction between a cylindrical particle and a flat plate can be both horizontal and vertical. By considering these geometrical orientations, it can be shown that the effective area of interaction for sphere to flat surface is the ring of molecule’s element inside the interacting bodies.

##### A Horizontal Cylindrical Particle Approach

**Figure 4:**
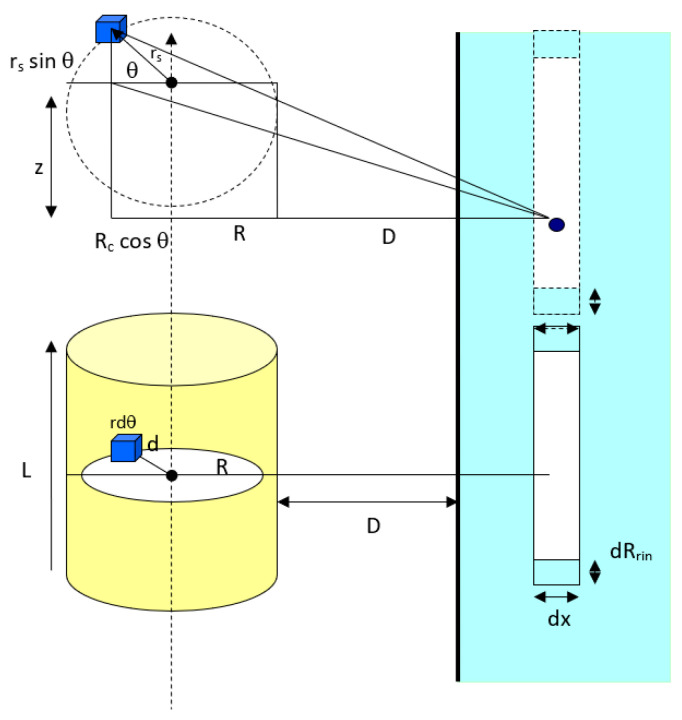
Interaction between a horizontal cylindrical particle and a solid flat surface.

As referred to Figure 4, the van der Waals interaction potential between a horizontal cylinder and a flat surface is the sum of all molecule elements in a cylinder of radius *R_c_* and length *L*. For an infinitesimal differential volume element of cross-sectional area, rdϕdr, and vertical height *dz*, the element volume is $r_{s}d\phi dr_{s}dz$ and the total number of molecules in the element is $\rho r_{s}d\phi dr_{s}dz$ where *ρ* is the number density of molecules in the cylinder. Thus, this enables us to calculate the overall interaction potential of molecules within the cylindrical particle. Geometrically, the distance between a differential volume element (the blue cube in Figure 4) and any molecule in ring in the flat surface is equal to:

(10) $$r=z^{2}+{\left(D+R_{c}+r_{s}\cos\varphi \right)}^{2}-r_{s}{}^{2}\sin^{2}\varphi$$

First, the interaction potential between the cylindrical particle (summation of overall molecules in the cylinder) to a molecule in ring in the flat surface will be:

(11) $$v\left(D\right)=-4C_{d}\rho_{c}\rho_{surf}\int_{0}^{\pi }\int_{0}^{Rc}\int_{0}^{L/2}\left(\frac{dz}{{\left[\left(z^{2}+{\left(D+R_{c}+r\cos\varphi \right)}^{2}\right)\pm r^{2}\sin^{2}\varphi \right]}^{3}}\right)rdrd\varphi$$

Second, to calculate the total van der Waals interaction potential between a horizontal cylinder and the ring in the flat surface as shown in Figure 4, the interaction of a cylinder and the circular element ring of cross-sectional area *πR_ring_*^2^ and thickness *dx* in the surface is considered. The volume of the ring is *πR_ring_*^2^*dx* and the number of molecules in this volume element is *ρ_surf_πR_ring_*^2^*dx*, where *ρ_surf_* is the number density of molecules in the flat surface. Since all the molecules in the ring are at the same separation distance *(D + x)* away from the cylinder surface, the overall van der Waals interaction potential between this element ring and the cylinder is *v*(*D* + *x*)*ρ_surf_ πR_ring_*^2^*dx*, where *v*(*D*) is given in Equation (11). Thus, the total interaction potential between the horizontal cylindrical particle and the flat surface is equal to:

(12) $$V_{vdW}\left(D+x\right)=-4\pi C_{d}\rho_{c}\rho_{surf}\int_{x=0}^{x=x}\left[v\left(D\right)\right]R_{ring}^{2}xdx$$

##### A Vertical Cylindrical Particle Approach

**Figure 5:**
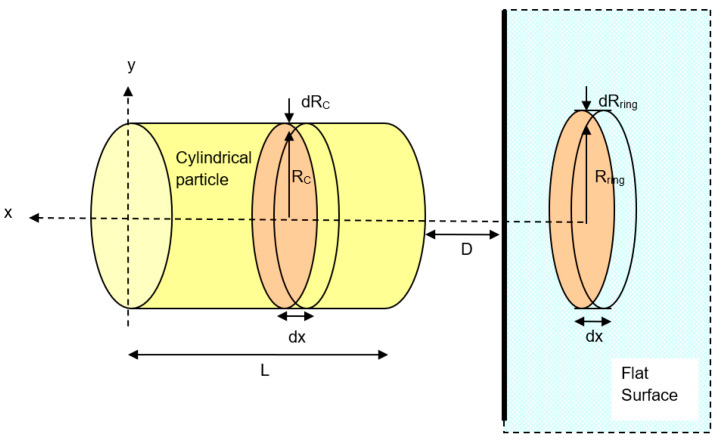
Interaction between a vertical cylindrical particle and a solid flat surface.

The total dispersion interaction for a vertical cylinder can be found in the same way as that for the spherical particle interaction, using the ring of elements from a slice of the cylindrical particle’s cross-sectional area (Figure 5). Except for *dx*, where *x* is changed from 0 to the cylinder length, *L*. From the volume of the cylinder, *V = πR_c_*^2^*L*; the total dispersion interaction potential for a vertical cylinder can be given as:

(13) $$V\left(D\right)=-\frac{\pi^{2}C_{d}\rho_{c}\rho_{surf}L}{6}\int_{x=0}^{x=L}\frac{\left(2R_{cyl}-x\right)x}{{\left(D+x\right)}^{3}}dx$$

#### 2.2.4. Retardation Effect in van der Waals Interaction

The derived van der Waals interaction potential equations for the geometrical shapes described earlier are unretarded (without retardation effect) interaction equations for sphere, cylinder, and rod-to-solid flat surfaces and are derived based on an implicit assumption that the speed of light is infinite. “Retardation effect” is the correction factor that must be made because of the reduction in the van der Waals interaction potential due to the finite speed of light [26,36]. Owing to the electromagnetic nature of the interaction, the actual van der Waals potential between two interacting bodies is reduced at large separation distances because the time it takes for the electric field to propagate from one body to another and back is such that the fluctuating electric moments become slightly out of phase. Such an effect may become very pronounced for macroscopic bodies at separation distances larger than about 5 nm [24]. For a large particle with diameter of 1 µm or greater, omission of the retardation has proved to lead to a serious overestimate of the van der Waals interaction[19]. In principle, the Hamaker approach can be modified to account for this retardation by multiplying the unretarded vdW interaction potential by a correction factor:

(14) $$v\left(r\right)=-\frac{C}{r^{n}}f\left(p\right)$$

**Figure 6:**
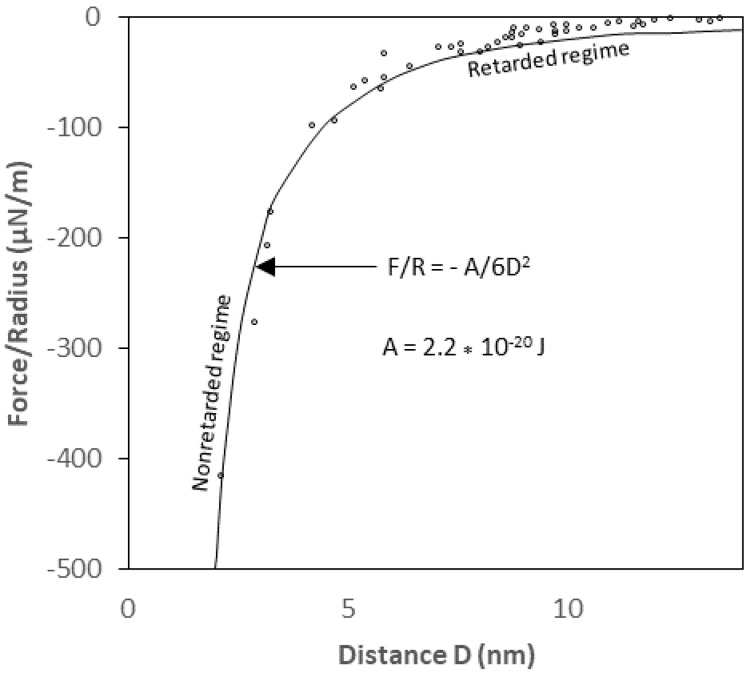
The van der Waals forces with non-retarded and retarded regimes [23].

Where the correction factor, *f*(*p*), depends on the reduced distance, $p=\;\frac{2\pi r}{\lambda }$, and *λ* is the “London characteristic wavelength” of the interaction. *λ* is assumed to be about 100 nm for most materials and the retardation only becomes significant when the separation distance between particles is of the same order as the characteristic wavelength [19] as shown in Figure 6. Since the rod particles are so much smaller than the flat plate, the existing correction factor for the sphere-plate interaction potential may be most suitable to account for the retardation effect on the rod-flat plate interaction system. The correction factor given by [37] as reported by [19] is as follows:

(15) $$f\left(D,\lambda \right)=\frac{\lambda }{\lambda -sD}$$

where, *λ* is a retardation parameter and “*s*” is a constant (s = 11.116). Multiplying the unretarded van der Waals interaction potentials by *f (D, λ)* yields the approximate retarded van der Waals interaction potential. When using a bacterium as the macroscopic body, there is only a small difference (up to 10–30J) between retarded and non-retarded vdW forces, and therefore will be ignored in this study.

### 2.3. Modeling Method and Software

The van der Waals interaction potentials for the interaction between bacteria and minerals can now be modeled for different geometrical conditions as a function of separation distance in producing a prediction of bacteria-mineral interaction.

The four geometrical conditions studied are:

1. Spherical shell bacteria to spherical mineral particle;
2. Horizontal capsule shaped bacteria to flat-plate mineral surface;
3. Vertical capsule shaped bacteria to flat-plate mineral surface;
4. Spherical shell bacteria to semi-infinite mineral wall.

(16) $$E_{vdW}=\;\frac{AR_{1}R_{2}}{6\left(R_{1}+R_{2}\right)}\;\left(\frac{1}{d+h_{1}+h_{2}}-\;\frac{1}{d+h_{2}}-\;\frac{1}{d+h_{1}}+\;\frac{1}{d}\right)-\frac{A}{6}\ln\left(\frac{d\left(d+h_{1}+h_{2}\right)}{\left(d+h_{1}\right)\left(d+h_{2}\right)}\right)$$

(17) $$E_{vdW}=\;\frac{AR}{6}\left(\frac{1}{d}-\frac{1}{d+h}\right)-\frac{A}{6}\ln\left(\frac{d}{\left(d+h\right)}\right)$$

The value of Hamaker constant, A, for different mineral interactions with bacteria are the values obtained from our previous investigation (Table 1) [38]. The calculations are made for both expressions at various separation distances, *D*, between 0 and 10 nm.

## 3. Results and Discussion

### 3.1. Capsule-Flat Plate Interaction Potential

As the van der Waals, vdW, interaction potentials of spherical and cylindrical particles to the flat plate have now been derived, the interaction potential between a capsule-like particle and a flat plate can be calculated by combining those two particle shapes interaction potentials. What follows describes the derived equations for the van der Walls interactions in vertical and horizontal approaches of a capsule-shaped particle.

#### 3.1.1. A Vertical Capsule-shaped Particle Approach

**Figure 7:**
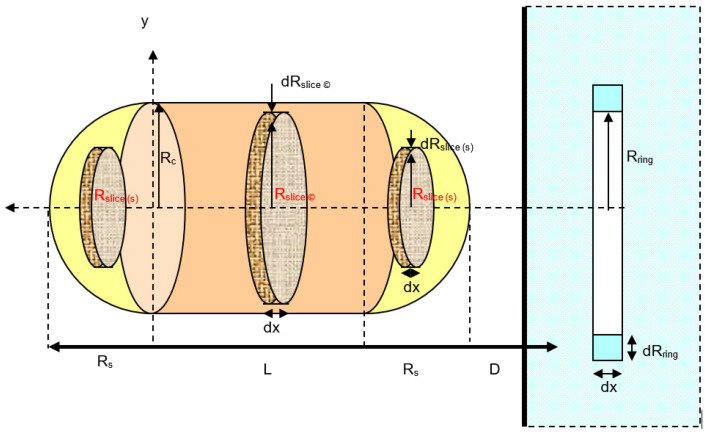
A schematic diagram for the interaction between vertical capsule and a solid flat plate.

As observed from Figure 7, for the interaction between a capsular particle and a flat plate, it is assumed that the particle is built from a combination of spherical and cylindrical particles with the same radius, (*R_c_* = *R_s_*). So that, for the vertical orientation capsule particle to the flat plate interaction as shown in Figure 7, the total van der Waals interaction potential becomes:

(18) $$V_{vdW}\left(H.Rod\right)=-\frac{\pi^{2}C\rho_{c}\rho_{surf}}{6}\left(\int_{0}^{2R_{sph}}\frac{\left(2R_{sph}-x\right)x}{{\left(D+x\right)}^{3}}dx+L\int_{0}^{L}\frac{\left(2R_{c}-x\right)x}{{\left(D+x\right)}^{3}}dx\right)$$

#### 3.1.2. A Horizontal Capsule-shaped Particle Approach

**Figure 8:**
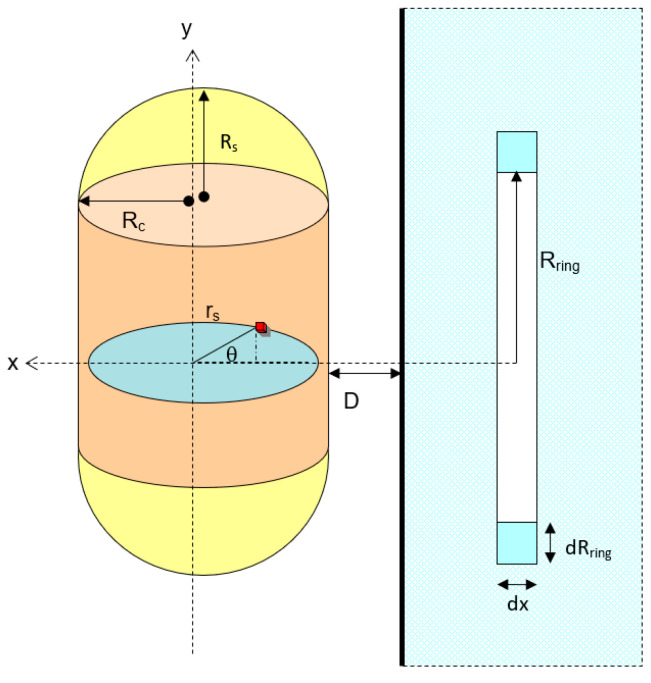
Schematic diagram for the interaction between vertical capsule and a solid flat plate.

When a capsule particle is horizontally positioned to the solid flat plate (Figure 8), the van der Waals interaction can be expressed as the combination between horizontal cylinder and the spherical particle as shown in Figure 8. Therefore, the total interaction potential is:

(19) $$V_{vdW}\left(Rod\right)=-\frac{\pi^{2}C_{d}\rho_{c}\rho_{surf}}{6}\int_{0}^{2R_{sph}}\frac{\left(2R_{sph}-x\right)x}{{\left(D+x\right)}^{3}}dx+-4\pi C_{d}\rho_{c}\rho_{surf}\int_{x=0}^{x=R_{c}}\left[v\left(D\right)\right]R_{ring}^{2}xdx$$

In order to obtain the van der Waals interaction potential, Equations (18) and (19) were used to calculate the van der Waals potential numerically using Maple software for both horizontal and vertical capsule shaped bacteria interactions to a mineral plate with a function of separation difference. The results will be compared with the conventional geometries applied for the van der Waals interaction between a particle and a surface.

### 3.2. Hamaker Interaction Constant for the Capsule-Shaped Particle

The Hamaker constant for interaction between two bodies is defined as [25,28]:

(20) $$A_{cs}=\pi^{2}C_{d}\rho_{c}\rho_{s}.\;$$

where *A_cs_* is the effective Hamaker constant for the interaction between phases (bacteria and a mineral surface) in vacuum. The parameter *C_d_* is the interaction coefficient and are the density of both interaction bodies. By inserting the Hamaker constant into each of the rod interaction equations, the new expressions become:

(21) $$V_{vdW}\left(H.Rod\right)=-\frac{A}{6}\left(\int_{0}^{2R_{sph}}\frac{\left(2R_{sph}-x\right)x}{{\left(D+x\right)}^{3}}dx+L\int_{0}^{L}\frac{\left(2R_{c}-x\right)x}{{\left(D+x\right)}^{3}}dx\right)$$

And,

(22) $$V_{vdW}\left(Rod\right)=-\frac{A}{6}\int_{0}^{2R_{sph}}\frac{\left(2R_{sph}-x\right)x}{{\left(D+x\right)}^{3}}dx+-4\frac{A}{\pi }\int_{x=0}^{x=R_{c}}\left[v\left(D\right)\right]R_{ring}^{2}xdx$$

The value of Hamaker constant, *A*, for different mineral interactions with bacteria are the values obtained in Table 1. The calculations are made for both expressions at various separation distances, *D*, between 0 and 10 nm.

### 3.3. The van der Waals Interaction Potential of P. Putida to Hematite and Quartz

**Figure 9:**
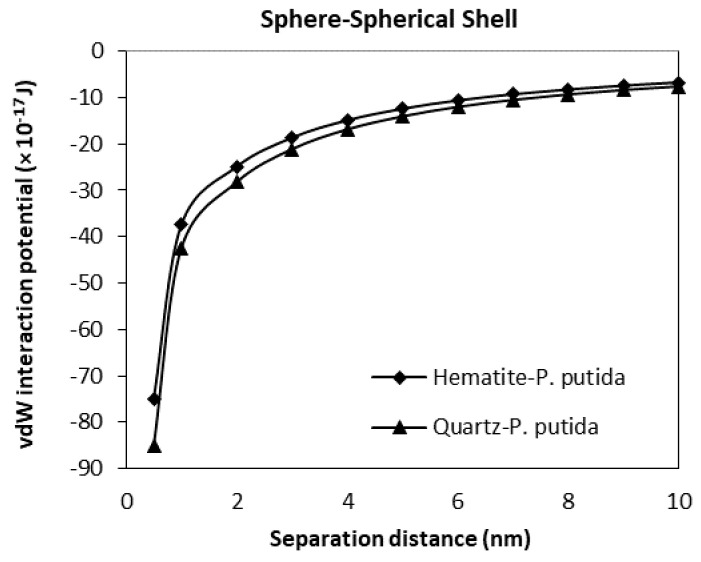
The van der Waals interaction energies of spherical (shell) bacteria approaching a spherical mineral particle surface.

**Figure 10:**
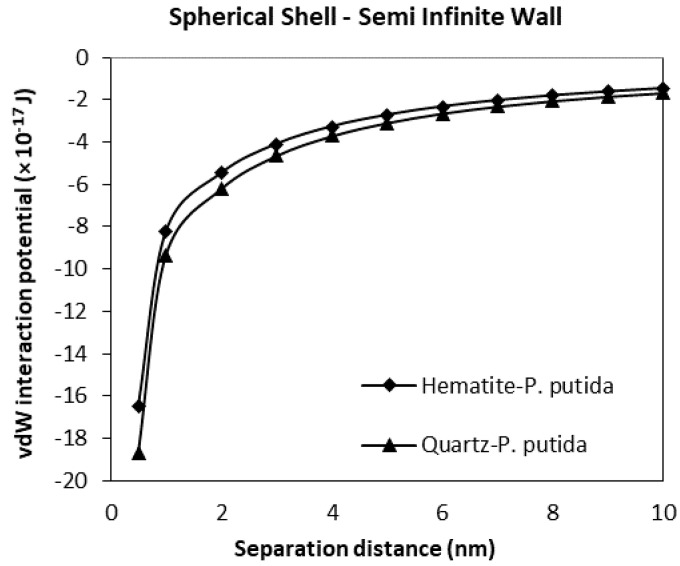
The van der Waals interaction energies of spherical shell bacteria approaching a semi-infinite mineral wall.

**Figure 11:**
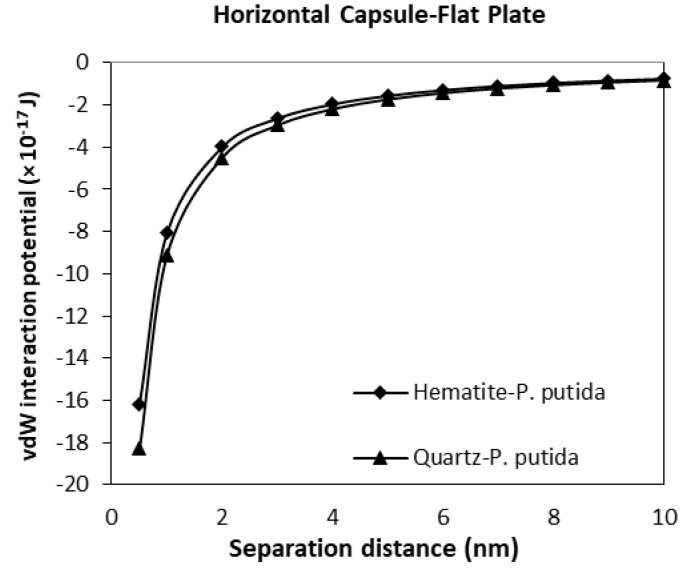
The van der Waals interaction energies of horizontal capsule shaped bacteria approaching a flat mineral plate.

**Figure 12:**
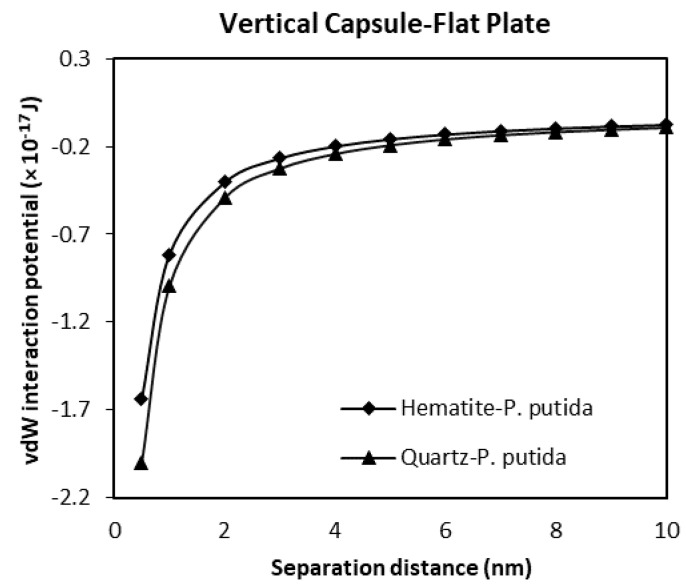
The van der Waals interaction energies of vertical capsule shaped bacteria approaching a flat mineral plate.

The van der Waals interaction potential of different shaped (geometry) and positioned bacteria to hematite and quartz flat plate surfaces have been calculated and plotted in Figure 9, Figure 10, Figure 11 and Figure 12. Results for each case of bacteria to the hematite and quartz were calculated from equations of vdW interaction potential for the spherical shell-spherical particle (sphere-sphere) interaction, and for the spherical shell-semi-infinite wall (sphere-plate) interaction (Table 1) and are plotted in Figure 9 and Figure 10 respectively. Figure 9 represents the plot of the van der Waals, (vdW) interaction potential for a spherical shell bacterium approaching a spherical mineral particle from a 10 nm to 0 nm separation distance. The vdW interaction potential of quartz-*P. putida* is higher than that of hematite, indicating a slightly more attractive potential of about 10 × 10^−17^ *J* between the two curves.

From Figure 10, at a separation distance from 10 nm to 0 nm, the vdW interaction potential curve is observed for the spherical shell bacteria to semi-infinite mineral wall. Similar to the spherical shell to spherical particle results, the attractive interaction between quartz and *P. putida* was more than hematite-*P. putida* interaction. However, compared to Figure 9, the vdW interaction potential of spherical shell-semi-infinite wall (at 0.5 nm, VvdW(D) for quartz is −18.7 × 10^−17^ J) is far smaller than that of the interaction between the spherical shell and spherical particle (at 0.05 nm, VvdW(D) for quartz is −85 × 10^−17^ J).

The interaction potential plots for a horizontal capsule bacterium to a flat plate mineral are given in Figure 11. Low potential is evident for both mineral types with the vdW interaction potential value of −16.2 (10^−17^ J) for hematite-*P putida*, and −18.3 (10^−17^ J) for quartz-*P. putida* interactions at a separation distance of 0.5 nm.

A much less negative and much smaller vdW attraction interaction potential is found for the interaction of vertical capsule shaped bacteria to a flat mineral plate (Figure 12). The maximum attraction potential for vertical capsule shaped *P. putida* to hematite interaction is about −1.64 × 10^−17^ J, while the quartz-*P putida* interaction is −2.0 × 10^−17^ J at a 0.5 nm separation distance.

From all four geometrical condition plots, it can be concluded that the vdW interaction potential of interactions between *P. Putida* and quartz is larger in magnitude than that of hematite-*P. putida* interactions. This indicates that *P. putida* is more attractive to quartz surface due to the current van der Waals interaction potential (but only for van der Waals interaction energy).

### 3.4. The Effect of Geometrical Shape to the van der Waals Interaction Potentials

**Figure 13:**
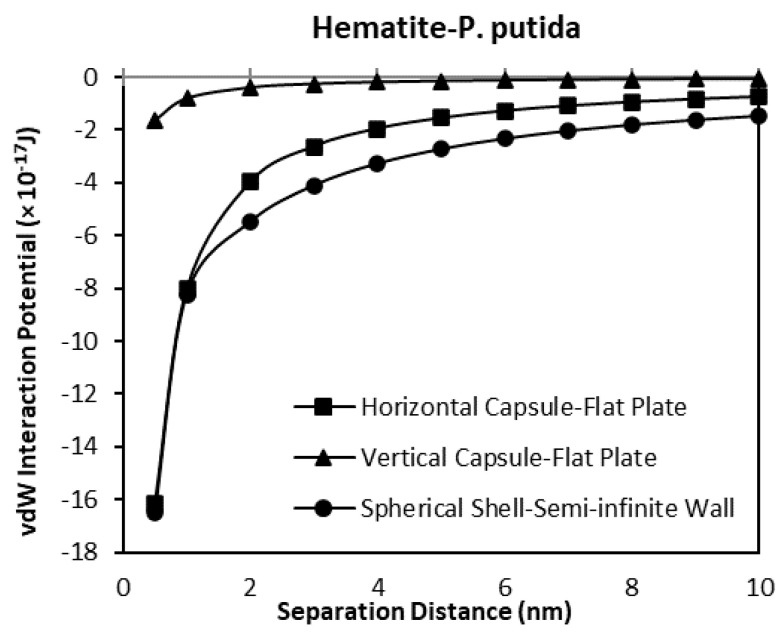
The van der Waals interaction potential of various *P. putida* to flat hematite plate.

**Figure 14:**
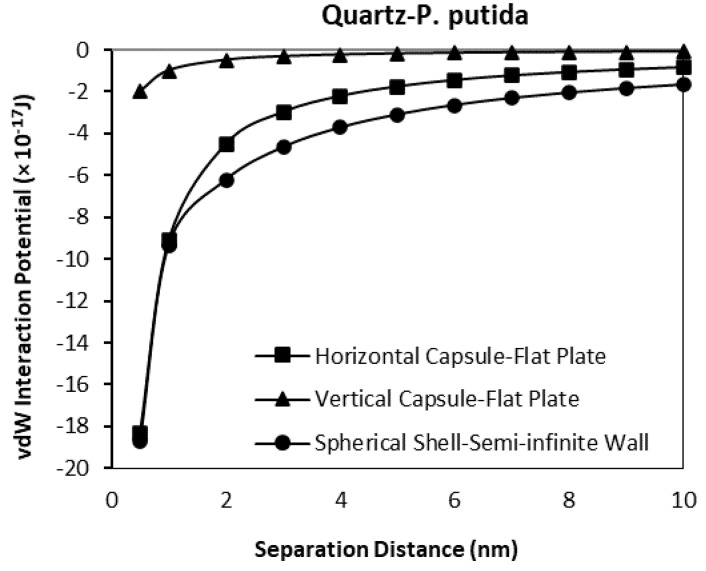
van der Waals interaction potential of various P. putida to flat quartz plate.

As a verification of the validity of the developed model, a comparison between the results obtained for horizontal and vertical capsule shaped bacteria are plotted along with the van der Waals expressions for spherical shell to semi-infinite wall developed by Tadmor [30]. Two plots for hematite and quartz as a function of separation distance are shown in Figure 13 and Figure 14 respectively. For these comparisons, the spherical shell bacteria to a spherical particle interaction are not included for the moment. The plots indicate the sensitivity of the van der Waals interaction potential to the geometrical shape of the bacteria. As the bacteria approach the mineral surface, the vdW becomes more negative indicating an attraction interaction between the bacteria and mineral surface. The geometry of the vertical capsule shaped bacteria to flat plate mineral shows the least change while the horizontal capsule-shaped bacteria to flat plate mineral and spherical shell bacteria to semi-infinite mineral surface give similar van der Waals interaction potentials at very short distances but a slightly higher potential (more negative) at distances more than 1 nm.

Given the relative size of the bacteria (1 μm diameter) to the distance over which the potentials have been calculated (1 nm), it is most likely that a capsule shaped bacteria will be in either a horizontal or vertical position when approaching the surface, and from general SEM and other microscopical observations of bacteria on surface it is most likely to be in the horizontal position (the long axis of the bacterial body being parallel to the surface).

**Figure 15:**
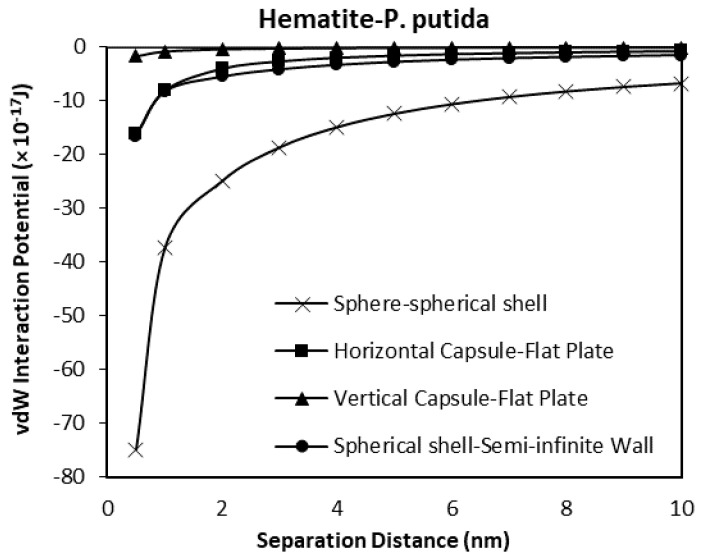
The van der Waals interaction energies of bacteria dispersed to hematite for different bacterial geometrical approach.

**Figure 16:**
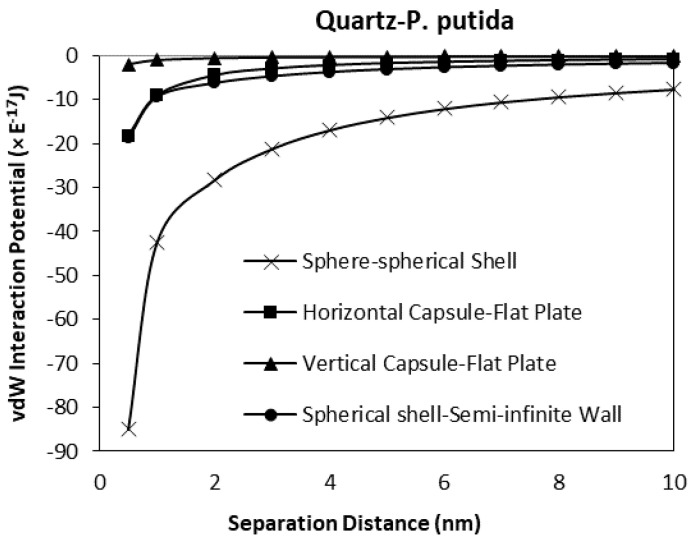
The van der Waals interaction energies of bacteria dispersed to quartz for different bacterial geometrical approach.

When the spherical shell bacteria to spherical mineral calculations are included in the comparison, the shape of the mineral surface has a far greater effect than the shape of the bacteria (Figure 15 and Figure 16). The spherical shell bacteria to spherical mineral shows maximum negative (i.e., attraction) potential values over the whole distance range. As the bacterium moves closer to the surface, increasing negative potential (increasing difference) is observed. At the closest distance calculated (0.5 nm), the van der Waals, vdW interaction potential is −74.9 × 10^−17^ J, rather than a maximum value of −18.3 × 10^−17^ J for the other calculations.

Thus, even when there is a large difference in size (bacterium as a sphere with radius of 0.5 μm, and mineral sphere is 0.5 cm radius, i.e., 1000× difference), a greater effect is obtained due to the spherical nature of the surface rather than the capsular shape of the bacteria.

Different minerals have different crystal shapes according to their atomic structure. Thus, when they exist as finely divided particles (e.g., in soils, water or on surfaces), they will present in a variety of shape forms to approaching bacteria. While a few will tend to crack as infinite flat plates (mica, etc.), most will have some form of angularity. Thus, it is most likely that the shape of the particle will dominate the van der Waals effect.

## 4. Conclusions

A general method is developed in this study to evaluate the van der Waals (vdW) interactions between a capsular particle (capsule shaped bacterium) to a flat plate in different approach profiles: Vertically and horizontally. A comparison of these approaches to the well-developed spherical particle to mineral surface (semi-infinite wall and spherical) approach was made. The van der Waals, vdW, interaction potentials for capsule-shaped bacterium were obtained using Hamaker’s microscopic approach of sphere-to-plate and cylinder-to-plate either vertically or horizontally to the flat surface. The numerical results show that a horizontal orientated capsule shaped bacterium to mineral surface interaction was more attractive compared to a capsule shaped bacterium approaching vertically. However, the vdW interaction potential obtained for spherical shell-sphere was very much more attractive (more negative) compared to the other three cases. From the study, it can be concluded that the effect of these conditions on the van der Waals interaction energy are:

1. The density difference between each type of bacterial shape (capsule, cylinder, and sphere) require different amounts of energy to adhere to hematite and quartz surfaces;
2. The orientations of bacteria approaching the mineral surface influences the adhesion of the cells to the mineral surface;
3. The type of mineral has an effect. In the case of *P. putida*, adherence is stronger to quartz than hematite;
4. The geometrical shape and curvature effect of the bacterial and mineral surfaces shows a greater interaction between spheres and spherical shell than other geometries and thus particle surface topography will have a considerable influence on the settlement of bacteria.

## Figures and Tables

**Table 1:** The Hamaker constants and the estimated geometric parameters for the bacteria and minerals.

Surface	*Pseudomonas putida*	Hematite	Quartz
Hamaker Constant, *A*	N/A	9.91 × 10^−20^	1.22 × 10^−19^
Sphere radius*, R_sphere_*	0.5 µm	-	-
Cylinder radius*, R_cylinder_*	0.5 µm	-	-
Shell thickness, *h_c_*	0.2 µm	-	-
Length of cylinder, *L*	2.0 µm	-	-
Mineral Radius:			
Radius of ring, *R_Ring_*	-	0.5 cm	0.5 cm
Separation Distance, *D*	-	0.5–10 nm	

## Data Availability

Data is contained within the article.
